# Stability and Efficacy of Chlorinated Disinfectants in Beninese Hospitals: Issues for the Prevention and Control of Infections and Antibiotic Resistance

**DOI:** 10.3390/tropicalmed11010012

**Published:** 2025-12-31

**Authors:** Sènami Evelyne Soclo Dansi, Comlan Cyriaque Degbey, Alphonse Kpozehouen, Nicolas Gaffan, Affi Diane Agbokou, Ounoussa Tapha, Dona Euloge Saïzonou, Houénoukpo Henri Soclo, Honoré Sourou Bankolé

**Affiliations:** 1 Clinique Universitaire d’Hygiène Hospitalière (CUHH), Centre National Hospitalier Universitaire—Hubert Koutoukou Maga (CNHU-HKM), Cotonou 01 BP 386, Benin; comlancy@yahoo.fr (C.C.D.);; 2 Institut Régional de Santé Publique (IRSP)—Comlan Alfred QUENUM, Université d’Abomey-Calavi (UAC), Ouidah 01 BP 384, Benin; 3 École Polytechnique d’Abomey-Calavi (EPAC), Université d’Abomey-Calavi, Abomey-Calavi 01 BP 2009, Benin

**Keywords:** chlorine solutions, bactericidal efficacy, infection prevention and control, university hospitals, Benin

## Abstract

In hospitals with limited resources, chlorine solutions are commonly used for biocleaning. The effectiveness of these solutions depends on the concentration of active chlorine and how they are prepared and stored. A study conducted in six University Hospitals in Benin from 10 March to 11 July 2025 aimed to evaluate the stability of active chlorine and the bactericidal efficacy of chlorine solutions used for disinfecting hospital environments. A total of 103 samples were analyzed using iodometric titration following the AFNOR (Association Française de Normalisation) standard NF EN ISO 7393-3 (2000) and WHO (World Health Organization) recommendations. Bactericidal activity was tested on multi-resistant hospital strains using the germ carrier method based on the standard NF T72-281. The study revealed that 88.4% of the solutions had inadequate active chlorine concentrations. Overall, the bactericidal activity was low, with only 14.6% effectiveness compared to 85.4% ineffectiveness. Ineffectiveness was particularly pronounced against Gram-negative bacilli, with 79.6% ineffectiveness and 20.4% effectiveness. Similarly, Gram-positive cocci showed a high level of ineffectiveness, reaching 84.5%, corresponding to 15.5% effectiveness. A significant association was observed between compliance with active chlorine concentrations and bactericidal effectiveness, with an OR of 42.5 and a *p*-value below 0.000001. Factors contributing to inefficiency included storage without light protection, use of transparent containers, storage for more than two days, inadequate active chlorine concentration, and incorrect pH levels. These issues compromise hospital disinfection and contribute to the persistence of multi-resistant bacteria in the hospital environment.

## 1. Background

Healthcare-associated infections (HAIs) are a significant global public health concern. They pose a threat to patient safety and contribute to higher rates of illness and death in hospitals. The WHO reports that 7–10% of patients in low- and middle-income countries develop HAIs during their hospital stay, a much higher rate than in developed countries [1]. In sub-Saharan Africa, the average prevalence of HAIs ranges from 12% to 15%, with higher rates observed in surgical, neonatal, and intensive care units [2,3]. Infections are often caused by lapses in infection prevention and control (IPC) practices, such as insufficient disinfection of surfaces and medical devices.

In many African settings, chlorinated solutions are commonly used as the primary disinfectant due to their broad antimicrobial coverage, affordability, and easy availability. Gallandat et al. showed that in resource-limited epidemic scenarios, chlorinated solutions are one of the most accessible and effective agents against a variety of pathogens [4]. Similarly, a study in 2024 reported that it is possible to produce chlorinated solutions locally with minimal cost while respecting microbiological efficacy [5]. However, several studies have shown that the efficacy of chlorine solutions is highly dependent on active chlorine concentration, pH, preparation method, and storage conditions [6,7]. The chemical instability of sodium or calcium hypochlorite, accentuated by heat, light, and time, frequently leads to rapid loss of disinfectant activity, reducing their bactericidal and virucidal efficacy [8]. However, several studies have shown that their effectiveness is highly dependent on active chlorine concentration, pH, preparation, and storage conditions [6,7]. The chemical instability of sodium or calcium hypochlorite, accentuated by heat, light, and time, frequently leads to rapid loss of disinfectant activity, reducing their bactericidal and virucidal efficacy [8].

In Benin, despite improvements in IPC, HAIs remain a significant challenge. Surveys conducted at University Hospitals (CHU “Centre Hospitalier Universitaire”) have revealed concerning rates of surgical site infections and neonatal infections, particularly at CNHU-HKM and CHUD-Borgou (Centre Hospitalier Universitaire Départemental de Borgou) [9,10]. Additionally, multidrug-resistant bacteria, primarily Gram-negative bacilli like *Klebsiella pneumoniae* and *Pseudomonas aeruginosa*, persist in the hospital environment in Benin [11]. These opportunistic pathogens are often found on surfaces, equipment, and water sources, indicating inadequate or irregular disinfection practices. The potential inefficacy of disinfectants may indirectly contribute to the selection and spread of multidrug-resistant strains through microbial adaptation mechanisms [12].

Studies have shown that repeated exposure to sublethal concentrations of chlorinated disinfectants can lead to the overexpression of Resistance-Nodulation-Division (RND) efflux pumps, resulting in cross-resistance to antibiotics [13,14]. Recent experiments have confirmed that insufficiently dosed sodium hypochlorite can trigger the production of reactive oxygen species and the development of adaptive mutations that lead to stable multidrug resistance in bacteria [15,16].

The effectiveness of chlorine solutions depends on the quality of the product and adherence to good preparation, labeling, and storage practices. The Africa CDC (Centers for Disease Control and Prevention) and WHO recommend monitoring active chlorine levels in hospitals to ensure continuous disinfection [17,18]. Stability and bactericidal efficacy of disinfectant solutions are crucial for assessing infection prevention and control measures.

This study aimed to evaluate the stability of active chlorine and the bactericidal efficacy of chlorine solutions used in Benin’s University Hospitals and identify associated factors. It provides current data on compliance with practices and factors influencing disinfectant performance, guiding strategies to enhance infection prevention and control quality.

## 2. Materials and Methods

### 2.1. Study Design

The study was carried out in six CHU in Benin (CNHU-HKM, CHU-MEL “Centre Hospitalier Universitaire de la Mère et de l’Enfant Lagune”, CHUD-Borgou, CHUD-Ouémé, CHUZ-Abomey-Calavi “Centre Hospitalier Universitaire de Zone d’Abomey-Calavi”, and CHUZ-Sourou-Léré), spread from the south to the north of the country. It is part of an approach to assess the quality of chlorinated solutions used for biocleaning in healthcare departments, as part of infection prevention and control.

### 2.2. Study Design and Period

This was a multicenter observational cross-sectional study combining field observations with laboratory analyses, conducted over a four-month period from 10 March to 11 July 2025.

### 2.3. Eligibility Criteria

The study included chlorine-based solutions used for disinfecting surfaces, equipment, or premises. This included solutions prepared locally or from diluted commercial products. All clinical departments that used chlorine-based disinfectant solutions at the time of the survey were eligible, including sites where chlorine solutions were produced or reconstituted within each facility.

This study does not include chlorinated solutions with unidentified chemical composition and those lacking minimal traceability.

### 2.4. Sampling Method

The observation unit consisted of ready-to-use disinfectant solutions containing sodium or calcium hypochlorite, used for the disinfection of surfaces and medical devices. A non-probability convenience sampling method was applied. This approach enabled the inclusion of all clinical departments using chlorine-based solutions, as well as all production or reconstitution sites within each CHU, thereby ensuring broad coverage of practices related to the preparation, storage, and use of chlorine-based disinfectants. All samples were collected at the beginning of the biocleaning process.

### 2.5. Data Collection

Chlorine solutions were collected in sterile opaque vials and transported in an insulated cooler equipped with a thermometer to ensure continuous temperature monitoring. During transport, the temperature was maintained between 2 °C and 8 °C using frozen cold packs. Upon arrival, all samples were received and registered at the CUHH at CNHU-HKM for analysis. A standardized collection form was used to record the origin, active ingredient, preparation date, storage conditions, and traceability of each sample.

### 2.6. Laboratory Analysis

#### 2.6.1. Physico-Chemical Analysis

##### pH Measurement

The initial pH of the chlorine solutions was measured directly in the field using a portable multiparameter handheld pH meter (ProfiLine Multi 3320 SET 1, Xylem Analytics Germany GmbH, Weilheim, Germany), equipped with a SenTix® 41 electrode dedicated to pH measurement. The instrument was calibrated in accordance with the manufacturer’s recommendations, guaranteeing reliable measurements. Two interpretation zones were defined according to the following classifications:

– pH < 8.5 ≥ 11: non-compliant, corresponding to an unstable zone where active chlorine degrades more rapidly.
– pH ≥ 8.5 < 11: compliant, corresponding to an optimum disinfectant efficiency zone.

##### Dosing of Active Chlorine

The active chlorine concentration of hypochlorite solutions was determined by iodometric titration, in accordance with AFNOR standard NF EN ISO 7393-3 (2000) [19] and WHO recommendations (2017) [20]. This method is based on the release of iodine in an acid medium, followed by titration with sodium thiosulfate and a starch indicator.

A reagent blank assay was carried out at the beginning of each series of manipulations under the same experimental conditions. Each determination was carried out in duplicate, and the active chlorine concentration was calculated according to the formula [19]:

$$Active\;Cl=\frac{N*V*MCl*2}{Vsample}$$

where

– *N* = normality of sodium thiosulfate (mol/L);
– *V* = volume of thiosulfate consumed (L);
– *MCl* = molar mass of chlorine (g/mol);
– *Vsample* = sample volume of chlorine solution used (L).

#### 2.6.2. Assessment of Bactericidal Activity

Bactericidal activity was evaluated using the qualitative germ carrier method, in-spired by the AFNOR NF T72-281 standard [21] and adapted to real conditions of use. Tests were performed on seven multidrug-resistant hospital bacterial strains, including five Gram-negative bacilli (*Klebsiella pneumoniae*, *Escherichia coli*, *Enterobacter cloacae*, *Acinetobacter baumannii*, and *Pseudomonas aeruginosa*) and two Gram-positive cocci (*Staphylococcus aureus* and *Enterococcus faecalis*).

These strains exhibited known resistance profiles and were isolated from the hospital environment in the participating university hospitals. They were used to assess the bactericidal efficacy of chlorinated solutions under conditions close to routine clinical practice.

After contamination, carriers were exposed to the chlorinated solutions under real-life conditions of use for a contact time of 30 min. Following exposure, carriers were swabbed and incubated in nutrient broth at 37 °C for 24–48 h, then subcultured onto selective media.

### 2.7. Study Variables

#### 2.7.1. Dependent Variables

Two dependent variables were selected to assess the quality of the chlorine solutions used:

▪ **Active chlorine concentration**

This variable reflects the actual oxidizing agent content in each solution tested. It enables us to assess the compliance of the solutions with the thresholds required for effective disinfection. Active chlorine concentrations were therefore interpreted in relation to the WHO-recommended reference value of 0.5% for biocleaning of surfaces and medical devices. A tolerance margin of ±0.1% was tolerated. Concentrations were characterized as follows:

– <0.4%: Insufficient: non-compliant;
– 0.4–0.6%: Adequate: compliant;
– 0.6%: acceptable but must be monitored.

▪ **Bactericidal quality**

This variable defines the ability of chlorinated solutions to inactivate hospital bacterial strains under actual conditions of use. Bactericidal efficacy was interpreted qualitatively based on bacterial growth:

– Clear broth and no growth: efficacy confirmed;
– Cloudy broth and bacterial growth: bactericidal ineffectiveness.

Positive growth controls, sterility controls, and neutralization tests were systematically performed to ensure the reliability and validity of the results. As bacterial counts before and after exposure were not quantified, bactericidal efficacy was assessed qualitatively and not expressed as a log_10_ reduction.

#### 2.7.2. Independent Variables

The independent variables were chosen for their epidemiological significance and their ability to impact the effectiveness of chlorine solutions in hospital settings. These variables include the type of health facility, the specific hospital service, the kind of disinfectant, temperature, exposure to light, shelf life, container material, type of container, presence of a hermetic cap, and pH level.

### 2.8. Quality Assurance and Quality Control Measures

Quality assurance procedures were applied throughout the analytical process. For chlorine titration, each batch included verification of reagents, calibration checks of analytical materials, and duplicate measurements. Bactericidal activity tests were performed according to standardized protocols using reference strains and positive and negative controls. All analytical steps followed the laboratory’s standardized operating procedures.

### 2.9. Statistical Analyses

Statistical analyses were performed using Epi Info™ version 7.2.6.0 (Centers for Disease Control and Prevention, Atlanta, GA, USA) and Microsoft Excel 2019 (Microsoft Corporation, Redmond, WA, USA). Data were checked for consistency and completeness.

Descriptive analysis was used to characterize chlorine solution samples based on their institutional origins, storage arrangements, physicochemical properties, and bactericidal efficacy. Qualitative variables were presented as frequencies and percentages, while quantitative variables were summarized using mean, standard deviation, median, quartiles, minimum, maximum, and mode.

Bivariate analysis was performed to explore associations between sample characteristics and two primary outcomes: non-compliance of active chlorine concentration and bactericidal ineffectiveness. Chi^2^ or Fisher tests were used for categorical variables, and crude Odds Ratios (OR) with 95% confidence intervals (CI95) and *p*-values were calculated. Variables with a significant association (*p* < 0.05) were included in multivariate analysis. Two logistic regression models were developed to identify factors independently associated with insufficient active chlorine concentration and bactericidal ineffectiveness. Variable selection was based on statistical significance, coefficient stability, and epidemiological relevance. Results were presented as adjusted ORs with IC95 and *p*-values, with a significance threshold of 5%.

## 3. Results

Below are the results obtained, along with the corresponding statistical analyses, to emphasize the factors influencing the stability of active chlorine and the bactericidal efficacy of chlorine solutions used in the university hospitals of Benin.

### 3.1. Characteristics of Chlorine Solution Samples Collected

A total of 103 chlorine solution samples were collected from Benin’s CHU and characterized. CNHU-HKM was the most represented with (20 samples; 19.4%), followed by CHUD-Ouémé (19 samples; 18.5%) and CHUD-Borgou (17 samples; 16.5%). These samples were taken in various clinical departments, notably: medicine (25.2%), followed by pediatrics (13.6%), maternity (12.6%), and neonatology (10.7%). Sodium hypochlorite is the most widely used type of chlorine solution (72.8%), while calcium hypochlorite accounts for 27.2%. 79.6% (82) of these solutions were produced by health facilities, and 20.4% (21) by external suppliers (Table 1).

#### 3.1.1. Extrinsic Characteristics of Chlorine Solution Samples

Table 2 shows that the majority of chlorine solutions used in Benin’s university hospitals are exposed to light (72.8%), 67.96% are stored at unsuitable temperatures (≥25 °C), and 76.7% are kept for inappropriate lengths of time (>2 days). Although most containers are fitted with hermetic caps (78.6%), almost half are made of transparent plastic.

#### 3.1.2. Intrinsic Characteristics of Chlorine Solution Samples

Analysis of the data reveals significant shortcomings in the physico-chemical and bactericidal quality of chlorine solutions used in Benin’s university hospitals. Of the 103 samples studied, only 12 (11.7%) had a compliant active chlorine concentration (≥0.5% ± 0.1), while 91 (88.4%) were insufficiently concentrated, resulting in a non-compliance prevalence rate of 88.4%. About solution pH, 38.8% complied (pH between 8.5 and 11) with recommended standards, compared with 61.2% non-compliant. Effective bactericidal quality was observed in 15 (14.7%) samples, while 88% of the bacterial strains tested were ineffective, resulting in a prevalence rate of non-effective bactericidal quality of 85.4% (Table 3).

### 3.2. General Distribution of Active Chlorine Concentrations

The mean active ingredient concentration of the chlorine solutions analyzed was 0.1680%, with a standard deviation of 0.156. The minimum concentration observed was 0.0120%, while the maximum was 0.6000%. The median was 0.1000%, and the mode was 0.0130%.

Quartiles indicate that 25% of concentrations were below 0.0400%, while 75% remained below 0.2600%. The distribution was asymmetrical, with a significant spread towards the lower values.

Bacterial growth was observed in 85.4% of broth cultures. This trend was observed for both sodium hypochlorite (86.7%) and calcium hypochlorite (82.1%). Statistical analysis (χ^2^ = 0.0024; *p* = 0.96; OR = 0.97; 95% CI: 0.28–3.34) showed no significant difference between the efficacy of sodium hypochlorite and calcium hypochlorite solutions (Table 4).

### 3.3. Efficacy of Chlorine Solutions on the Bacterial Groups Tested

The bactericidal efficacy of the chlorinated solutions measured was 20.4% on Gram-negative bacilli (GNB) and 15.5% on Gram-positive cocci (PGC).

### 3.4. Factors Associated with Insufficient Active Chlorine Concentration

#### 3.4.1. Bivariate Analysis of Factors Associated with Insufficient Active Chlorine Concentration

The bivariate analysis showed that several basic sample characteristics were significantly associated with insufficient active chlorine concentration (Table 5). Chlorine solutions stored at a temperature ≥ 25 °C (COR = 4.3; 95% CI: 1.3–14.1), exposed to light (COR = 6.2; 95% CI: 1.7–22.4), stored in transparent containers (COR = 8.4; 95% CI: 1.9–36.7), with a storage duration > 2 days (COR = 6.9; 95% CI: 1.4–33.8), or with a non-compliant pH (COR = 8.2; 95% CI: 1.6–41.3) had significantly higher odds of presenting insufficient active chlorine concentration. These characteristics all showed high Crude Odds Ratios and statistically significant *p*-values, indicating their contribution to active chlorine degradation.

Other variables—including health facility, hospital department, type of chlorine compound, labeling of the container, type of storage container, and presence of a hermetic cap—showed no statistically significant association with insufficient active chlorine concentration (*p* > 0.05).

#### 3.4.2. Multivariate Analysis

Multivariable logistic regression (Table 6) identified several independent predictors of insufficient active chlorine concentration. Solutions stored with exposure to light showed a significantly higher likelihood of non-compliance (AOR = 6.2; 95% CI: 1.7–22.4; *p* = 0.006). Storage at temperatures ≥ 25 °C was also strongly associated with inadequate active chlorine levels (AOR = 4.3; 95% CI: 1.3–14.1; *p* = 0.015). Transparent containers markedly increased the odds of non-compliance compared with opaque containers (AOR = 8.4; 95% CI: 1.9–36.7; *p* = 0.003). In addition, storage duration exceeding recommended limits was associated with a higher risk of insufficient active chlorine concentration (AOR = 6.9; 95% CI: 1.4–33.8; *p* = 0.017). Finally, non-compliant pH values remained a strong independent predictor of chlorine degradation (AOR = 8.2; 95% CI: 1.6–41.3; *p* = 0.011).

### 3.5. Factors Associated with Bactericidal Ineffectiveness

#### 3.5.1. Bivariate Analysis of Factors Associated with Bactericidal Ineffectiveness

In bivariate analysis, five sample characteristics were significantly associated with ineffective bactericidal quality. Solutions stored without protection against light (*p* = 0.038), in non-opaque containers (*p* = 0.025), with inadequate storage time (*p* = 0.037), inadequate pH (*p* = 0.036), or insufficient active chlorine concentration (*p* = 0.011) were at greater risk of bactericidal ineffectiveness. No statistically significant association was observed between institutional variables (university hospital, hospital department), type of disinfectant, type of storage container, presence of hermetic cap, container labeling, or storage temperature and the bactericidal quality of chlorine solutions (*p* > 0.05) (Table 7).

#### 3.5.2. Multivariate Logistic Analysis

Multivariable logistic regression identified several independent factors significantly associated with the ineffective bactericidal quality of chlorine-based disinfectant solutions (Table 8). Solutions stored without protection from light were nearly five times more likely to exhibit inadequate bactericidal activity (AOR = 4.6; 95% CI: 1.2–17.9; *p* = 0.026). Storage in transparent containers was also strongly associated with reduced efficacy (AOR = 5.1; 95% CI: 1.3–20.2; *p* = 0.021). Furthermore, solutions stored for a non-compliant duration had a significantly higher likelihood of being ineffective (AOR = 4.4; 95% CI: 1.1–17.2; *p* = 0.035). A non-compliant pH was another independent predictor of poor bactericidal activity (AOR = 4.7; 95% CI: 1.2–18.3; *p* = 0.030). Finally, solutions that had a non-compliant active chlorine concentration were over seven times more likely to display insufficient bactericidal performance (AOR = 7.2; 95% CI: 1.5–34.1; *p* = 0.011).

Overall, these findings highlight the critical influence of storage conditions (light exposure, container opacity, and duration), physicochemical stability (pH), and active chlorine concentration on the bactericidal effectiveness of chlorine-based disinfectants.

## 4. Discussion

A study conducted in six CHUs in Benin identified substantial deficiencies in the quality of chlorine solutions used for hospital cleaning. Among the 103 samples analyzed, only 11.7% met the required active chlorine concentration, and 14.7% demonstrated adequate bactericidal efficacy. These findings reveal a high level of non-compliance, with 88.4% of samples failing physico-chemical standards and 85.4% failing microbiological performance criteria, raising important concerns for healthcare safety.

The study found that a concerning 88.4% of disinfectants in hospitals had insufficient active chlorine content. This raises significant concerns about the quality of biocleaning practices. Inadequate chlorine levels not only render the disinfectants ineffective but also promote the survival of bacteria in sublethal conditions. This can lead to the development of adaptive mechanisms that result in bacterial tolerance and multi-resistance.

Repeated or prolonged exposure of microorganisms to low doses of chlorinated disinfectants, such as sodium hypochlorite, can cause the development of adaptive mechanisms that may result in cross-resistance to antibiotics. In a study by Nam et al., it was demonstrated that exposure to low doses of sodium hypochlorite can cause an increase in the expression of RND (Resistance-Nodulation-Division) efflux pumps in *Pseudomonas aeruginosa*. This overexpression results in decreased susceptibility to imipenem and other β-lactam antibiotics [13]. Efflux pumps, which are linked to reduced membrane permeability, are a crucial mechanism for biocide tolerance and contribute to the spread of multidrug-resistant bacteria [16]. Similarly, Aljuwayd et al. [15] demonstrated that sublethal exposure to chlorine causes increased production of reactive oxygen species (ROS), leading to adaptive genetic mutations and secondary antibiotic resistance in Salmonella [14]. Wu-Chen et al. also confirm that prolonged exposure to food-grade disinfectants promotes stable cross-resistance to several classes of antibiotics, including fluoroquinolones and β-lactams [22].

These observations support previous findings, indicating that inadequately dosed biocides can create selective pressure similar to antibiotics [14]. This pressure can trigger bacterial adaptive responses, including the activation of efflux systems, modification of intracellular targets, and DNA repair mechanisms. Improper use or insufficient application of disinfectants in hospitals may undermine the efficacy of bio-cleaning and ultimately promote the development and persistence of multidrug-resistant strains in the hospital setting.

Our findings indicate that while chlorine solution remains the predominant disinfectant in university hospitals, its efficacy is highly dependent on how it is prepared and applied. The connection between inadequate disinfection and bacterial resistance is indirect but well-documented: using insufficient disinfectant can create a selective environment that promotes the development of more resistant bacterial strains.

Several factors contribute to these non-conformities, including exposure to light, storage temperature of 25 °C or higher, use of transparent containers, prolonged storage exceeding 2 days, and non-compliant pH levels. These factors were found to be statistically associated with the degradation of active chlorine and reduced bactericidal effectiveness. This highlights the importance of proper storage conditions in maintaining the stability and efficacy of disinfectant solutions. Similar findings have been reported in hospitals in Uganda, Tanzania, and Nigeria, where non-compliance rates ranging from 60% to 90% have been observed [7,23,24].

A multivariate analysis identified several factors linked to insufficient active chlorine concentration in chlorine solutions used in Benin University hospitals. These factors included inadequate light protection during storage, high storage temperatures (≥25 °C), use of transparent containers, prolonged storage times (>2 days), and incorrect pH levels. All of these variables were found to be statistically significant, highlighting the influence of storage conditions and physico-chemical properties on active chlorine stability. These findings are consistent with previous studies that have demonstrated the rapid degradation of active chlorine due to photodegradation and oxidation of hypochlorite [6,25,26]. In the tropical conditions of Benin, high temperatures and frequent exposure to light exacerbate these phenomena, reducing the active life of disinfectant solutions [8]. In hot and humid conditions like those found in Benin’s university hospitals, ready-to-use solutions should not be stored for more than 24 h, following WHO guidelines [27].

The pH imbalance in some solutions is a key factor in their instability. Research indicates that sodium hypochlorite breaks down faster when the pH is below 8.5 or above 12, resulting in a rapid decline in active chlorine and the creation of less effective by-products [28]. To extend the stability and bactericidal effectiveness of chlorine solutions, it is essential to adjust the pH and carefully manage storage conditions [8].

Out of the solutions tested, only 14.6% showed satisfactory bactericidal efficacy based on the WHO’s defined thresholds. This finding is similar to a study conducted in Uganda in 2024 [7], where most locally prepared solutions lost their microbicidal activity after a few days of storage.

A strong correlation was found between the concentration of active chlorine and bactericidal efficacy. Compliant solutions were far more likely to be effective than non-compliant ones, further underscoring the importance of maintaining proper active chlorine levels.

Similarly, String et al. showed that an improperly dosed chlorine solution can lose up to 90% of its effectiveness on contaminated surfaces. This is especially true in cases of excessive dilution or prolonged storage [29].

The study findings reveal issues that extend beyond just the technical aspects of chlorine solution quality. In Benin’s University Hospitals, the main obstacle to IPC is not just the availability of equipment but also the need for strict adherence to protocols, a culture of accountability, and raising awareness among all staff members.

The study found that 85.44% of the chlorine solutions analyzed were not effective at killing bacteria, posing a significant risk of incomplete disinfection of surfaces and medical devices. This could potentially lead to the development of multi-resistant bacteria. In Benin, there is a high prevalence of HAIs in referral hospitals. A national survey conducted by Ahoyo et al. in 2014 reported an overall HAI prevalence of 19.1%, with urinary, pulmonary, and surgical site infections being the most common types [10].

A study conducted at CNHU-HKM in Cotonou by Dégbey et al. found a 7.81% prevalence of surgical site infections, which was closely associated with asepsis and disinfection practices [9].

Several African studies have highlighted the importance of biocleaning in preventing neonatal infections. For instance, a multicenter study in sub-Saharan Africa by Nakibuuka et al. found that confirmed neonatal infection rates were between 28% and 35%, mainly due to insufficient cleaning practices and the use of improperly disinfected shared equipment [30]. A study conducted in nine public hospitals in Benin found that standard hospital hygiene precautions are not adequately followed, leading to an increase in neonatal infections, especially in intensive care units [31]. These results highlight the critical importance of strengthening disinfection protocols and providing staff training in these departments. The data, along with our findings, suggest that the ineffectiveness of disinfectants due to chemical and microbiological non-conformity may contribute to the rise in multi-resistant bacteria and the persistence of hospital-acquired infections. This means that hospitals can unknowingly facilitate the spread of diseases when disinfection products fail to work correctly.

## 5. Limits

While these results are significant, it is essential to acknowledge some methodological limitations and recognize the strengths of this study.

This study is the first national evaluation of the stability and effectiveness of chlorine solutions in Benin’s university hospitals. It followed a standardized methodology, including WHO-recommended tests, and was conducted in six representative university hospitals. This comprehensive approach offers an unbiased assessment of disinfectant quality in Benin’s healthcare facilities. Additionally, the rigorous statistical analysis identified key factors associated with non-compliance, providing valuable insights for national infection prevention and control strategies.

However, there are limitations to consider for a comprehensive interpretation of the findings. The study was cross-sectional so that it could not track the degradation of active chlorine over time. Storage conditions varied among hospitals, and organizational factors like staff training and supervision were not explored. Furthermore, certain organizational determinants, such as staff training modalities, supervision practices, and work organization, may influence the performance of chlorine solutions without having been specifically analyzed in this study. Lastly, while the study demonstrated a loss of bactericidal efficacy, its direct impact on healthcare-associated infections and bacterial resistance was not assessed.

## 6. Conclusions

This study in Benin’s hospitals was the first to assess the stability and bactericidal efficacy of chlorine solutions nationwide. The research revealed significant variations in active chlorine concentration, directly impacting the effectiveness of disinfection. Exposure to sublethal doses of chlorine can lead to the development of adaptive mechanisms in microorganisms, such as increased efflux pump expression, which can contribute to bacterial multi-resistance. These findings emphasize the importance of monitoring active chlorine levels regularly and implementing better practices for preparing, storing, and using disinfectants in hospitals. This is essential to prevent a decrease in disinfection effectiveness and the potential emergence of antimicrobial cross-resistance.

## Figures and Tables

**Table 1:** Characteristics of chlorinated water solution samples (*n* = 103).

Variables	Frequency	Percentage
Hospitals		
CNHU/CHU-MEL	36	35.0
CHUD_Borg/Ouémé	36	35.0
CHUZ_Ab-Calavi/Sou-Léré	31	30.1
Hospital services		
Technical platforms/interventions	32	31.1
General care/maintenance	33	32.0
Mother/child	38	36.9
Type of disinfectant		
Sodium hypochlorite	75	72.8
Calcium hypochlorite	28	27.2

**Table 2:** Storage and preservation characteristics of chlorinated solutions (*n* = 103).

Variables	Frequency	Percentage
Store away from light.		
Yes	28	27.2
No	75	72.8
Temperature < 25 °C		
Yes	33	32.0
No	70	68.0
Storage container		
Seal	49	47.6
Can	54	52.4
Container material		
Transparent plastic	50	48.5
Opaque plastic	53	51.5
Presence of an airtight cap		
Yes	81	78.6
No	22	21.4
Storage duration		
Compliant	24	23.3
Not compliant	79	76.7

**Table 3:** Physico-chemical and bactericidal characteristics of chlorine solution samples (*n* = 103).

Variables	Frequency	Percentage
Active chlorine concentration		
Adequate—compliant	12	11.7
Insufficient—non-compliant	91	88.4
Solution pH		
Compliant	40	38.8
Non-compliant	63	61.2
Bactericidal quality		
Effective	15	14.6
Non-effective	88	85.4

**Table 4:** Efficiency rates by type of chlorine solution.

Type of Disinfectant	Development of Bacteria (%)		
	No	Yes	Total
Calcium hypochlorite	5 (17.9)	23 (82.1)	28 (27.2)
Sodium hypochlorite	10 (13.3)	65 (86.7)	75 (72.8)
Total	15 (14.6)	88 (85.4)	103 (100.0)

**Table 5:** Association between insufficient active chlorine concentration in chlorine solutions and basic sample characteristics.

Variables	Insufficient Active Chlorine Concentration			
	Not Compliant %	Crude OR	95% CI	*p*-Value
Health facilities				
CHUD_Borg/Ouémé	88.2%	1.0	—	—
CNHU/CHU-MEL	94.6%	2.1	0.6–7.3	0.239
CHUZ_Ab-Calavi/Sou-Léré	93.5%	1.9	0.5–7.1	0.312
Services				
Labeling	91.1%	1.0	—	—
Mother and child	88.9%	0.8	0.2–3.2	0.744
Technical platforms/interventions	94.4%	1.7	0.4–7.3	0.459
Type of disinfectant				
Calcium hypochlorite	88.2%	1.0	—	—
Sodium hypochlorite	93.3%	1.9	0.5–6.9	0.319
Storage protected from light				
Yes	74.2%	1.0	—	—
No	94.4%	6.2	1.7–22.4	0.006
Temperature < 25 °C				
Yes	75.9	1.0	—	—
No	93.2	4.3	1.3–14.1	0.015
Container correctly labeled				
Yes	92.0	1.0	—	—
No	93.8	1.3	0.3–5.7	0.739
Storage container				
Can	91.7	1.0	—	—
Seal	93.3	1.2	0.3–5.0	0.803
Container material				
Opaque	77.3	1.0	—	—
Transparent	96.6	8.4	1.9–36.7	0.003
Presence of hermetic cap				
Yes	91.5	1.0	—	—
No	94.4	1.6	0.4–6.7	0.518
Storage duration				
Compliant	75.0	1	—	—
Non-compliant	95.1	6.9	1.4–33.8	0.017
pH value				
Compliant	66.7	1	—	—
Non-compliant	94.7	8.2	1.6–41.3	0.011

**Table 6:** Multivariable logistic regression analysis of factors associated with insufficient active chlorine.

Variables	Adjusted OR	95% CI	*p*-Value
Storage protected from light			
Yes	1		
No	6.2	1.7–22.4	0.006
Temperature < 25 °C			
Yes	1		
No	4.3	1.3–14.1	0.015
Container material			
Opaque	1		
Transparent	8.4	1.9–36.7	0.003
Storage duration			
Compliant	1		
Non-compliant	6.9	1.4–33.8	0.017
pH value			
Compliant	1		
Non-compliant	8.2	1.6–41.3	0.011

**Table 7:** Bivariate analysis of factors associated with the bactericidal ineffectiveness of chlorine solutions.

Variables	Inefficacy (%)	OR Brut	95% CI	*p*-Value
Health facilities				
CHUD_Borg/Ouémé	37.8	1	-	-
CNHU/CHU-MEL	33.3	0.8	0.3–2.3	0.697
CHUZ_Ab-Calavi/Sou-Léré	42.9	1.3	0.4–4.2	0.647
Services				
Labeling	38.9	1	-	-
Mother and child	33.3	0.8	0.2–2.9	0.762
Technical platforms/interventions	41.7	1.1	0.3–4.2	0.878
Type of disinfectant				
Calcium hypochlorite	38.5	1	-	-
Sodium hypochlorite	37.5	1.0	0.4–2.6	0.964
Storage protected from light				
No	40.3	1	-	-
Yes	17.2	0.3	0.10–0.94	0.038
Temperature < 25 °C				
No	37.8	1	-	-
Yes	17.2	0.4	0.11–1.09	0.071
Opaque Container				
No	41.0	1	-	-
Yes	16.7	0.3	0.10–0.86	0.025
Storage duration				
Non-compliant	38.5	1	-	-
Compliant	14.3	0.3	0.08–0.93	0.037
Container correctly labeled				
No	40.0	1	-	-
Yes	36.4	0.9	0.3–2.6	0.838
Container type				
Seal	40.0	1	-	-
Can	36.4	0.9	0.3–2.6	0.838
Presence of an airtight cap				
No	40.0	1	-	-
Yes	36.4	0.9	0.3–2.6	0.838
pH interpretation				
Non-compliant	41.7	1	-	-
Compliant	16.7	0.3	0.09–0.93	0.036
Active chlorine concentration				
Non-compliant	39.6	1	-	-
Compliant	8.3	0.1	0.03–0.63	0.011

**Table 8:** Factors associated with ineffective bactericidal quality of chlorine solutions.

Variables	Adjusted OR	95% CI	*p*-Value
Storage protected from light			
Yes	1		
No	4.6	1.2–17.9	0.026
Container material			
Opaque	1		
Transparent	5.1	1.3–20.2	0.021
Storage duration			
Compliant	1		
Non-compliant	4.4	1.1–17.2	0.035
pH value			
Compliant	1		
Non-compliant	4.7	1.2–18.3	0.030
Active chlorine concentration			
Compliant	1		
Non-compliant	7.2	1.5–34.1	0.011

## Data Availability

The data supporting the findings of this study are not publicly available due to ethical and confidentiality restrictions related to hospital-based microbiological data but are available from the corresponding author upon reasonable request.

## Abbreviations

The following abbreviations are used in this manuscript (alphabetically ordered):

AFNOR	Association Française de Normalisation (French Standardization Association)
CDC	Centers for Disease Control and Prevention
CHU	Centre Hospitalier Universitaire (University Hospital)
CHUD	Centre Hospitalier Universitaire Départemental (Departmental University Teaching Hospital)
CHU-MEL	Centre Hospitalier Universitaire de la Mère et de l’Enfant Lagune (Lagoon Mother and Child University Hospital Center)
CHUZ	Centre Hospitalier Universitaire de Zone (Zonal University Hospital)
CI	Confidence Interval
CNHU-HKM	Centre National Hospitalier Universitaire Hubert Koutoukou Maga (Hubert Koutoukou Maga National University Hospital Center)
CLERB-UP	Comité Local d’Éthique pour la Recherche Biomédicale de l’Université de Parakou (Local Ethics Committee for Biomedical Research of the University of Parakou)
CUH	Clinique Universitaire d’Hygiène Hospitalière (University Clinic for Hospital Hygiene)
Epi Info™	Epidemiological analysis software developed by the CDC
HAI	Healthcare-Associated Infection (Infections associées aux soins, IAS)
IPC	Infection Prevention and Control
NF EN ISO	Norme Française/Européenne/Internationale d’Organisation de Normalisation (French/European/International Standardization Standard)
OR	Odds Ratio
pH	Hydrogen Potential
T72-281	AFNOR Standard related to airborne disinfection processes
WHO	World Health Organization
